# Sodium Selenite Enhances Antibiotics Sensitivity of *Pseudomonas aeruginosa* and Deceases Its Pathogenicity by Inducing Oxidative Stress and Inhibiting Quorum Sensing System

**DOI:** 10.3390/antiox10121873

**Published:** 2021-11-24

**Authors:** Weina Kong, Qianqian Tian, Qiaoli Yang, Yu Liu, Gongting Wang, Yanjun Cao, Liping Wang, Sizhe Xia, Yanmei Sun, Cheng Zhao, Shiwei Wang

**Affiliations:** 1 Key Laboratory of Resources Biology and Biotechnology in Western China, Ministry of Education, The College of Life Sciences, Northwest University, Xi’an 710069, China; kongwn@nwu.edu.cn (W.K.); tianqianqian@stumail.nwu.edu.cn (Q.T.); yangqiaoli@stumail.nwu.edu.cn (Q.Y.); liuyu1@stumail.nwu.edu.cn (Y.L.); wanggongting@stumail.nwu.edu.cn (G.W.); cao2014@nwu.edu.cn (Y.C.); wangliping@stumail.nwu.edu.cn (L.W.); xiasizhe@stumail.nwu.edu.cn (S.X.); sunyanmei@nwu.edu.cn (Y.S.); zhaocheng@stumail.nwu.edu.cn (C.Z.); 2 Provincial Key Laboratory of Biotechnology of Shaanxi Province, College of Life Sciences, Northwest University, Xi’an 710069, China

**Keywords:** *Pseudomonas aeruginosa*, sodium selenite, antibiotic susceptibility, quorum sensing, ROS, oxidative stress

## Abstract

*Pseudomonas aeruginosa*, a Gram-negative opportunistic pathogen, is commonly found in clinical settings and immuno-compromised patients. It is difficult to be eradicated due to its strong antibiotic resistance, and novel inactivation strategies have yet to be developed. Selenium is an essential microelement for humans and has been widely used in dietary supplement and chemoprevention therapy. In this study, the physiological and biochemical effects of sodium selenite on *P. aeruginosa* PAO1 were investigated. The results showed that 0~5 mM sodium selenite did not impact the growth of PAO1, but increased the lethality rate of PAO1 with antibiotics or H_2_O_2_ treatment and the antibiotics susceptibility both in planktonic and biofilm states. In addition, sodium selenite significantly reduced the expression of quorum sensing genes and inhibited various virulence factors of this bacterium, including pyocyanin production, bacterial motilities, and the type III secretion system. Further investigation found that the content of ROS in cells was significantly increased and the expression levels of most genes involved in oxidative stress were up-regulated, which indicated that sodium selenite induced oxidative stress. The RNA-seq result confirmed the phenotypes of virulence attenuation and the expression of quorum sensing and antioxidant-related genes. The assays of Chinese cabbage and *Drosophila melanogaster* infection models showed that the combination of sodium selenite and antibiotics significantly alleviated the infection of PAO1. In summary, the results revealed that sodium selenite induced oxidative stress and inhibited the quorum sensing system of *P. aeruginosa*, which in turn enhanced the antibiotic susceptibility and decreased the pathogenicity of this bacterium. These findings suggest that sodium selenite may be used as an effective strategy for adjunct treatment of the infections caused by *P. aeruginosa*.

## 1. Introduction

*Pseudomonas aeruginosa* is a ubiquitous Gram-negative opportunistic bacterium that can cause respiratory and urinary tract, burn, and wound infections [1,2]. It harbors a broad arsenal of virulence factors for infections establishment, including pyoverdine, pyocyanin, rhamnolipids, hemolysins, and proteases, and they largely contribute to bacterial pathogenesis [3]. In addition, biofilm is an important factor that causes bacterial antibiotic resistance. The widespread use of antibiotics leads to the increasing emergence and spread of multidrug-resistant strains (MDR), making the traditional antibiotic therapy against *P. aeruginosa* infection ineffective [4]. Thus, it is urgent to develop novel antibacterial agents for effectively treating MDR infections, such as phage therapy, nanoparticle therapy, and quorum sensing inhibitors [5].

Quorum sensing has been believed to be an excellent antibacterial drug target. It is involved in a process of cell-to-cell chemical communication and relies on bacterial production, detection, and response to extracellular signaling molecules called autoinducers [6]. Many ordinated behaviors of bacteria are regulated by the quorum sensing system, such as bioluminescence [7], virulence factor production [8], secondary metabolite production [9], and biofilm formation [10]. Three quorum sensing systems have been found in *P. aeruginosa*, including *las*, *rhl*, and *pqs*. In the *las* and *rhl* systems, the signaling molecules are acyl-homoserine lactones, and for *pqs* it is 2-heptyl-3-hydroxy-4-quinolone. These quorum sensing systems coordinate the regulation of virulence factors and biofilm formation of *P. aeruginosa*, and it is thought that the breakdown of quorum sensing systems may lead to a significant reduction in bacterial pathogenicity [11]. Thus, the inhibition of quorum sensing systems can be a novel and effective way to fight this MDR bacterium.

Selenium is one of the trace elements necessary for human bodies and many organisms, and it was discovered by Swedish chemist Jons Jacob Berzelius in 1817. Initially, selenium was considered to be toxic, while until the 1950s selenium was found to be a basic element of organisms [12]. Selenium has important biological functions and affects organisms in a dose-dependent manner. In the environment, it exists in many forms, such as selenate (Se^+6^), selenite (Se^+4^), elemental selenium (Se^0^), selenide (Se^−2^), selenocysteine, selenomethionine, etc. [13,14]. Selenium compounds can react with a thiol to form highly reactive and unstable metabolites, and these metabolites then react with oxygen and generate reactive oxygen species (ROS) [15], which can cause cell damage, such as protein and lipid peroxidation and DNA oxidation [16]. Among these selenium compounds, sodium selenite has long been regarded as one with the most redox activity to produce ROS and the most effective to kill cancer cells [17,18], and it has a strong ability to oxidize mercaptan substances, such as glutathione (GSH) and thioredoxin (Trx).

Although selenium has been well known as an antioxidant and anticancer agent [19] and also has been reported to have strong antibacterial activity against some planktonic cells of bacteria, such as *Bacillus subtilis*, *Staphylococcus aureus*, *Escherichia coli*, and *Klebsiella planticola* [20], the detailed effects of selenite on bacterial physiology remain to be explored. In this study, the low concentration of selenite (0.5~5 mM) was found to have no effects on *P. aeruginosa* growth, but obviously increased the lethality rate of this bacterium with antibiotics or H_2_O_2_ treatment. The antibiotic susceptibility in planktonic and biofilm states was both increased, and the bacterial virulence and pathogenicity were reduced. The detailed mechanisms were further investigated by bioinformatics and biochemical methods.

## 2. Materials and Methods

### 2.1. Bacterial Strains and Culture Conditions

The bacterial strains and plasmids used in this study are listed in Table 1. The *P. aeruginosa* strains were routinely cultivated at 37 °C in LB medium without sodium chloride (LBNS, peptone 10.0 g/L, yeast extract 5.0 g/L, pH 7.0). When necessary, antibiotics were used at the following concentrations: for *E. coli*, kanamycin (Kn) at 50 μg/mL; for *P. aeruginosa*, trimethoprim (Tmp) at 500 μg/mL. For growth curve detection, *P. aeruginosa* PAO1 was cultured in 5 mL LBNS broth at 37 °C and 200 rpm overnight, and 50 μL of culture was transferred into 5 mL of fresh LBNS broth with 0, 0.5, 1, 5, 10, and 20 mM sodium selenite and incubated at 37 °C for 12 h. The absorbance value at 600 nm (OD_600_) of the culture was measured by ultraviolet spectrophotometry. The medium for swarming motility examination consisted of 0.8% nutrient broth (Oxoid, Basingstoke, UK), 0.5% d-glucose, and 0.5% agar [21]. The swimming motility medium was comprised of 0.8% nutrient broth, 0.5% d-glucose, and 0.3% agar. Thin LBNS plates with 1% agar were used for twitching motility assay.

### 2.2. Survival Assays of Antimicrobial Treatment

The survival of PAO1 with various antimicrobial challenges was assayed as described previously with minor modification [22]. In brief, after culture overnight, 200 µL aliquots were transferred to 5 mL fresh LBNS medium with different antibiotics that have no significant effect on the growth of PAO1 (including 2 μg/mL gentamicin, 0.5 μg/mL tobramycin, 15 μg/mL ampicillin, 20 ng/mL ciprofloxacin, or 9 μg/mL tetracycline) and a final concentration of 5 mM sodium selenite. As regards the H_2_O_2_ challenge, a final concentration of 3 mM H_2_O_2_ was used under the condition with or without 5 mM sodium selenite. After 1 h, 2 h, 3 h, or 4 h growth, the cultures were diluted serially and plated on LBNS agar plates at 37 °C overnight. The colony-forming units (CFUs) were counted and the survival rates were calculated.

### 2.3. Determination of Minimal Inhibitory Concentration (MIC), Minimal Biofilm Inhibitory Concentration (MBIC), and Minimal Biofilm Eradication Concentration (MBEC) with the Combination of Sodium Selenite and Antibiotics

MIC of antibiotics with or without sodium selenite was detected by the broth microdilution method as previously described [23]. Briefly, gentamicin and tobramycin ranging from 0.25 to 64 μg/mL were prepared using 2-fold serial dilutions in 96-well polyvinyl chloride plates (Corning/Costar, Corning, NY, USA) with 100 μL of LBNS broth per well, respectively. Selenite was added into parallel wells to obtain 1- or 5-mM final concentrations, and no selenite samples were detected as controls. Then, 1 μL of overnight cultures of *P. aeruginosa* PAO1 were added and the plates were incubated at 37 °C for 12 h. OD_600_ was measured. The lowest concentration of antibiotics with or without selenite that inhibited bacterial growth until OD_600_ < 0.1 after 12 h incubation was taken as MIC. The measurement of MBIC and MBEC was performed on 96-well plates as described above. MBIC represented the minimum concentration of antibiotics and selenite that lead to biofilm formation with OD_560_ < 0.1, and MBEC was the minimum concentration of antibiotics and selenite that eliminated the 10-h biofilm (OD_560_ < 0.1). MBIC was detected after static growth for 12 h at 30 °C. MBEC was determined after 12 h of treatment to the formed biofilm by different concentrations of antibiotics and selenite.

### 2.4. Biofilm Formation Measurement

Biofilm formation was measured in a static system as previously described [24]. In brief, overnight cultures were diluted at 1:100 in a fresh LBNS medium, and 100 μL of the medium was transferred into 96-well polyvinyl chloride plate. Sodium selenite was added until a final concentration of 5 mM. After incubation at 37 °C for 5 h without shaking, the free-floating planktonic cells were removed and wells were washed three times with distilled water. Biofilms were stained with 120 μL of 0.1% crystal violet for 20 min, and each well was washed with water to remove unbound dye. The remaining crystal violet was dissolved in 200 μL of 30% glacial acetic acid and 125 μL of the solution was transferred to the 96-well microtiter plate. The absorbance at 560 nm (OD_560_) was measured.

### 2.5. Pyocyanin Production Assay

The assay of pyocyanin production was carried out according to the method described previously with slight modifications [25]. Briefly, overnight cultures were inoculated at 1:100 dilutions into 5 mL fresh LBNS broth with or without 5 mM sodium selenite and incubated at 37 °C for 48 h. Pyocyanin was extracted from the culture supernatants by adding 3 mL of chloroform. After extraction, the chloroform layer was transferred into a new tube and mixed with 1 mL of 0.2 M HCl. The top layer was removed after centrifugation and the absorbance was measured at 520 nm (OD_520_). Concentrations, expressed as µg of pyocyanin produced per mL of culture supernatant, were determined by multiplying OD_520_ by 17.072 [26].

### 2.6. Bacterial Motility Examination

For swarming and swimming examination, 2 µL of overnight culture was inoculated onto plates at 37 °C for 12 h. Twitching motility was assayed by stab-inoculating bacteria through the thin LBNS agar plates at 30 °C for 24 h under humidified conditions. Sodium selenite was used until a final concentration of 5 mM. Photographs were taken using a Tanon 2500 imaging system and the diameter of the movement circle was measured. Each experiment was repeated three times.

### 2.7. Rhamnolipid Quantification

Rhamnolipid was measured using the modified colorimetric anthrone assay [27]. In brief, bacteria were cultured for 48 h in nutrient broth with or without 5 mM sodium selenite, and 2 mL of fermentation liquid were centrifuged to extract the supernatant. An aliquot of 400 μL anthrone (0.2% anthrone in 85% H_2_SO_4_) was added to 100 μL supernatant and heated at 100 °C for 30 min. The mixture was allowed to cool to room temperature and the absorbance at 620 nm (OD_620_) was measured. Rhamnolipid levels in the supernatant were calculated according to the standard curve, which was obtained with rhamnose standards (Solarbio, Beijing, China).

### 2.8. Construction of the Promoter-Reporter Plasmids

The plasmid pMS402 was used to construct gene expression vectors as reported previously [28]. The promoter regions of target genes were respectively amplified by PCR using the corresponding primers (Table 1). The PCR products were cloned into the *Bam*HI-*Xho*I site upstream of the *lux* genes on pMS402. The constructed plasmids were transformed into *E. coli* DH5α competent cells and the positive clones were screened out by 50 μg/mL kanamycin and DNA sequencing.

### 2.9. Gene Expression Analysis

Gene expression of *lux*-based reporters in liquid cultures was measured as counts per second (cps) of light production in a Synergy H2 Plate Reader (Biotek, Winooski, VT, USA). Overnight cultures of the reporter strains were diluted at 1:20 and incubated for an additional 3 h. The cultures were inoculated into parallel wells of a black 96-well plate with a transparent bottom. A volume of 10 µL of the fresh cultures were inoculated into the wells containing 90 μL of LBNS medium with or without 5 mM sodium selenite. Forty microliters of filter-sterilized mineral oil was added to prevent evaporation during the assay. Bacterial growth and promoter activities were measured in the microplate reader every 2 h intervals for 24 h.

### 2.10. Measurement of Intracellular ROS Level

Cell pellets of PAO1 from 500 μL of overnight culture were harvested, and stained with 500 μL of 20 μM 2′,7′-dichlorofluorescein diacetate (carboxy-H_2_DCFDA, dissolved in sterile PBS) at 37 °C for 40 min under the dark condition. The treated cells were collected and washed with 500 µL of sterile PBS three times to fully remove the carboxy-H_2_DCFDA that did not bind. The cells were resuspended in 500 µL of PBS with or without 5 mM sodium selenite and then incubate at 37 °C in dark for 2 h, and 4 mM H_2_O_2_ was used as a positive control. The cells were centrifuged, washed three times, and resuspended in PBS. A volume of 200 µL of suspension was transferred into a black 96-well plate with transparent bottom and the fluorescence was measured using the microplate reader with emission at 528 nm and excitation at 485 nm. The fluorescence data were normalized to the cell density of each sample. Three independent experiments were performed.

### 2.11. Superoxide Dismutase (SOD) Activity Assay

Overnight cultures were transferred at 1:100 into 10 mL of fresh LBNS liquid with or without 5 mM and cultivated to an OD_600_ of 1.0. A volume of 2 mL of bacterial culture was harvested by centrifugation, washed twice, and finally resuspended in 1 mL of pre-cooling PBS. After Cells were lysed by ultrasonic crushing apparatus and centrifuged at 8000× *g* rpm for 5 min, the supernatant was collected and the activity of SOD was measured using the SOD activity detection kit (Beyotime, Shanghai, China). The enzyme activity was normalized to the cell density of each sample. Three independently repeated experiments were carried out.

### 2.12. RNA-Seq Sample Preparation and Sequencing

The overnight culture was transferred to a fresh LBNS medium with or without 5 mM sodium selenite and incubated to an OD_600_ = 0.8. The collected cells were washed once with PBS. The bacterial cell pellets were quickly frozen with liquid nitrogen before being sent to Hangzhou Lianchuan Biotechnology (Co. Ltd., Hangzhou, China) for total RNA extraction, cDNA library construction, and RNA-seq analysis. RNA-Seq was performed on the Illumina HiSeq™ 2000 (San Diego, CA, USA). The raw paired-end reads were trimmed and quality controlled by Trimmomatic with parameters (version 0.36 http://www.usadellab.org/cms/uploads/supplementary/Trimmomatic (accessed on 9 October 2020). Then clean reads were separately aligned to reference genome with orientation mode using Rockhopper (http://cs.wellesley.edu/~btjaden/Rockhopper (accessed on 9 October 2020) software.

### 2.13. Bioinformatics Analysis

Differential expression genes (DEGs) of samples were detected using DESeq. The transcripts with log_2_fold change ≥1 and *P*_adj_ (*p*-value adjusted) ≤0.05 were considered to be DEGs. The COG (Cluster of Orthologous Group) database of proteins was analyzed by BLAST, and the COG functional annotation was performed. Kyoto Encyclopedia of Genes and Genomes (KEGG) pathways enrichment was analyzed by using tools in the DAVID web server [29]. The number of DEGs in different KEGG pathways was counted to signaling pathways.

### 2.14. Promoter Activity Assay

The GFP reporter plasmid pHERD20T was used to construct transcriptional fusions with the promoters of the genes *pqsR*, *lasR*, and *rhlR*. The promoter region was amplified by PCR. The DNA fragment was digested and cloned into the digested pHERD20 plasmid. The resulting plasmids were transferred into *P. aeruginosa* PAO1. The promoter activity was evaluated by measuring GFP intensity and OD_600_ in the microplate reader (Synergy H2, BioTek, Winooski, VT, USA).

### 2.15. Chinese Cabbage Infection Assay

A bacterial virulence test was performed as described previously with minor modifications [30]. *P. aeruginosa* PAO1 was grown in LBNS medium at 37 °C overnight. After centrifugation, the bacterial cells were washed in 10 mM MgSO_4_ and diluted to OD_600_ = 1.0, and then 10 μL of suspension was injected with a syringe into the midrib of Chinese cabbage leave after disinfection by 5% sodium hypochlorite and 70% ethanol. The leaves were placed on dishes containing a Whatman filter impregnated with 10 mM MgSO_4_. The plates were kept in an incubator at 37 °C, and rot symptoms were monitored every two days. Finally, the rotten part was cut off and soaked in 1 mL of PBS, and placed for 4 °C overnight. The cabbage was taken out and weighed, and the collected bacterial liquid was coated with a gradient dilution method to count the number of bacteria in the rotten part. The number of bacteria was expressed as CFU/mg. The experiments were repeated at least three times on independent periods.

### 2.16. Drosophila Melanogaster Infection Assay

*P. aeruginosa* PAO1 was grown overnight, washed, and diluted in 5% (*w*/*v*) sucrose to OD_600_ = 2.0. Groups of 10 male fruit-flies (five to seven days old) were starved for 3 h and then fed continuously at 25 °C in vials with sterile filter papers, which had been previously added with the bacterial solution. Fruit-fly survival was monitored daily. Three groups of 30 fruit-flies were used for each condition in three independent experiments.

### 2.17. Statistical Analysis

All reported experiments were independently repeated at least three times. Statistical analysis was carried out using GraphPad Prism 5 and values mean ± SD. The data were analyzed by one-way analysis of variance (ANOVA). A Student’s *t*-test was used when one-way ANOVA revealed significant differences (*p* < 0.05).

## 3. Results

### 3.1. Sodium Selenite Increased the Lethality Rate of P. aeruginosa PAO1 with Antibiotics or H_2_O_2_ Treatment and the Antibiotics Susceptibility in Planktonic and Biofilm States

**Figure 1:**
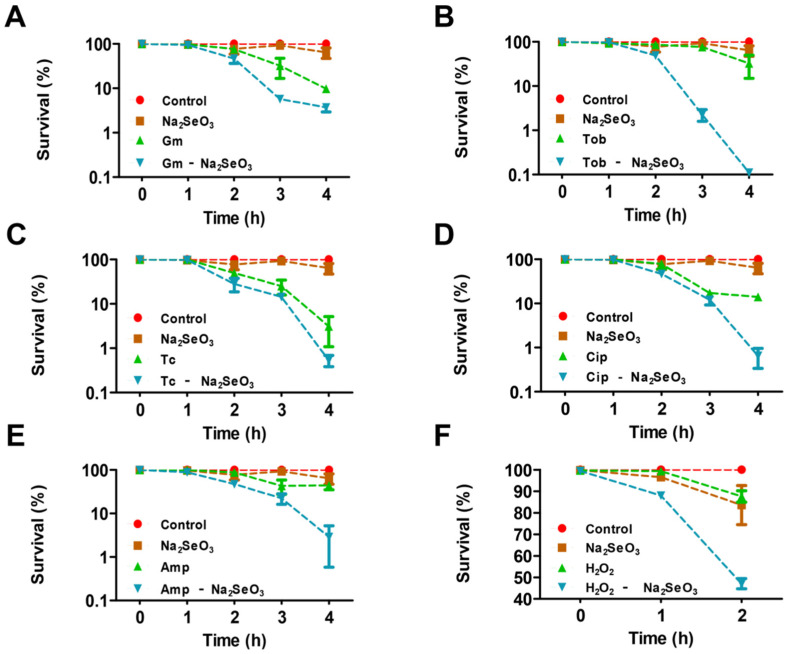
Evaluation of bacterial survival with antibiotics and sodium selenite treatment for various periods. (**A**) The survival rate of PAO1 treated with sodium selenite, gentamicin, or the combination of the two compounds. (**B**) The survival rate of PAO1 treated with sodium selenite, tobramycin, or the combination of the two compounds. (**C**) The survival rate of PAO1 treated with sodium selenite, tetracycline, and the combination of the two compounds. (**D**) The survival rate of PAO1 treated with sodium selenite, ciprofloxacin, or the combination of the two compounds. (**E**) The survival rate of PAO1 treated with sodium selenite, ampicillin, or the combination of the two compounds. (**F**) The survival rate of PAO1 treated with sodium selenite, H_2_O_2_, or a combination of the two compounds.

The growth of *P. aeruginosa* PAO1 was firstly examined in the presence of sodium selenite in this study. As reported previously [20], sodium selenite showed antibacterial activity when the concentrations were more than 10 mM. When the concentration of sodium selenite was less than 5 mM, it had no significant effect on PAO1 growth in LBNS liquid (Figure S1). Therefore, sodium selenite of 5 mM was selected for subsequent studies. Interestingly, the survival assay showed that 5 mM sodium selenite obviously reduced the survival rate of PAO1 in the presence of antibiotics (Figure 1A–E), especially for the aminoglycoside antibiotic tobramycin (Figure 1B), the fluoroquinolone antibiotic ciprofloxacin (Figure 1D), and the β-lactam antibiotic ampicillin (Figure 1E). As regards the oxidant H_2_O_2_, when 5 mM sodium selenite was combined to use, a similar result was obtained (Figure 1F). Taken together, the results showed that 5 mM sodium selenite increased the lethality rate of *P. aeruginosa* in the presence of antibiotics and oxidant agents.

**Figure 2:**
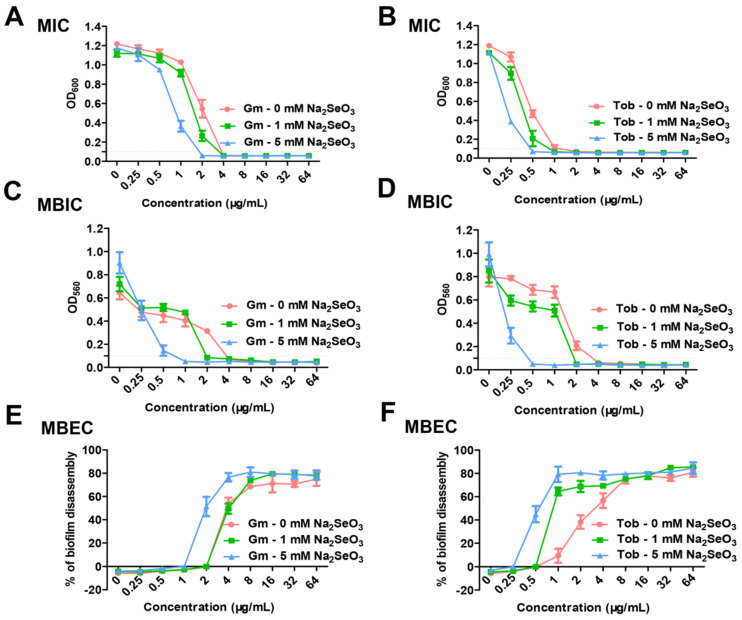
Effects of sodium selenite on the antibiotic susceptibility of PAO1. (**A**,**B**) PAO1 was treated with varying concentrations of gentamicin and tobramycin (0.25 to 64 μg/mL) in LBNS broth at 37 °C for 12 h. The lowest concentration of antibiotics and sodium selenite that inhibited bacterial growth (OD_600_ < 0.1) after 12 h incubation was taken as MIC. (**C**,**D**) PAO1 was treated with varying concentrations of gentamicin and tobramycin (0.25 to 64 μg/mL) in LBNS broth and static growth at 30 °C for 12 h. MBIC represents the minimum concentration of antibiotics and sodium selenite required to inhibit biofilm formation (OD_560_ < 0.1). (**E**,**F**) The 10-h biofilm of PAO1 was treated with varying concentrations of gentamicin and tobramycin (0.25 to 64 μg/mL) in LBNS broth and static growth at 30 °C for 12 h. All experiments were repeated independently three times.

Furthermore, MIC values of antibiotics with or without sodium selenite were determined. When 5 mM sodium selenite was used in combination with gentamicin or tobramycin, the MIC values of gentamicin or tobramycin were both remarkedly decreased (Figure 2A,B and Table S1), while 1 mM sodium selenite had no obvious influence on the MIC values of these two antibiotics (Figure 2A,B). The results indicated that sodium selenite could enhance the antibiotic susceptibility of *P. aeruginosa* PAO1.

It is known that biofilm formation increases the drug resistance of bacteria [31]. The effect of sodium selenite on the antibiotic susceptibility of *P. aeruginosa* in the biofilm state was further examined. The result showed that the MBIC value of gentamicin for 12-h static biofilm growth was 4 μg/mL, whereas combined with 5 mM sodium selenite, the MBIC value of gentamicin was reduced to 1 μg/mL (Figure 2C). The synergistic effect of sodium selenite with tobramycin also was observed, as the MBIC value of tobramycin was dramatically decreased from 4 μg/mL to 0.5 μg/mL (Figure 2D). In addition, the MBIC values of the two antibiotics were also obviously reduced in the presence of 1 mM sodium selenite.

To determine whether sodium selenite enhanced the ability of antibiotics to eradicate the formed biofilm, we examined the MBEC values of gentamicin and tobramycin to eradicate a 10-h biofilm. As shown in Figure 2E,F, the MBEC values of gentamicin and tobramycin with sodium selenite were two-fold lower than those without sodium selenite. These results demonstrated that sodium selenite increased the antibiotic susceptibility of bacteria in the biofilm state, indicating that sodium selenite could be coupled with antibiotic therapy to combat biofilm-related infections.

### 3.2. Sodium Selenite Inhibited Biofilm Formation, Pyocyanin Production, Bacterial Motilities, and the Type III Secretion System (T3SS)

**Figure 3:**
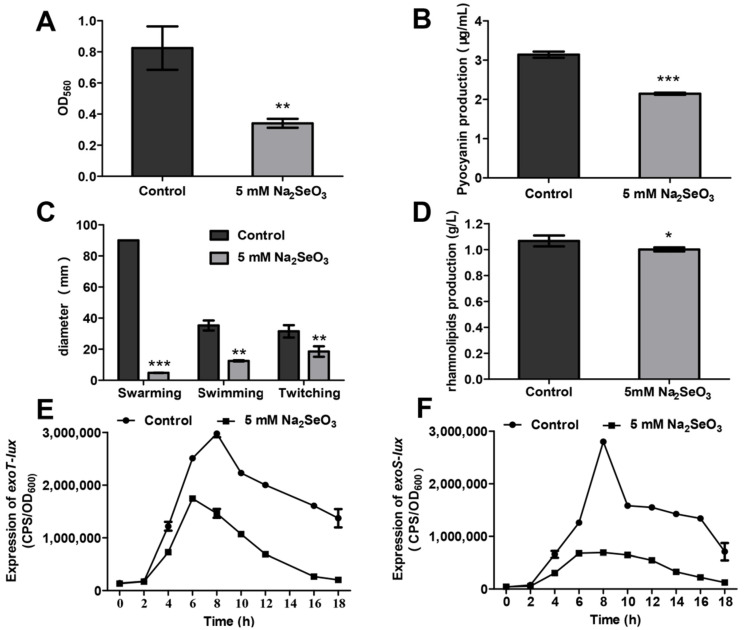
Sodium selenite affected biofilm formation (**A**), pyocyanin production (**B**), bacterial motilities (**C**), rhamnolipids production (**D**), and the expression of T3SS effector genes (**E**,**F**). For biofilm formation assay, the biomass of PAO1 was detected by CV staining after sodium selenite treatment. For the expression examination of T3SS effector genes, a CTX-*exoT* or CTX-*exoS* reporter fusion integrated on the chromosome was used to measure expression levels of T3SS genes under T3SS inducing conditions with or without sodium selenite after 18-h growth. All data were presented as mean ± SD of three separate experiments. * *p* < 0.05, ** *p* < 0.01, *** *p* < 0.001, compared with the untreated control group.

The effects of sodium selenite on the phenotypes related to the pathogenicity of *P. aeruginosa* were examined. The results showed that 5 mM sodium selenite inhibited the formation of biofilms (Figure 3A). The production of pyocyanin was decreased from 3.15 µg/mL to 2.15 µg/mL in the presence of 5 mM sodium selenite (Figure 3B). The motilities including swarming, swimming and twitching were all impaired by 5 mM sodium selenite (Figure 3C). Furthermore, the swarming motility of PAO1 was totally inhibited (Figure S2). In addition, the rhamnolipid level was decreased when 5 mM sodium selenite was added (Figure 3D). T3SS is the key virulence system relative to acute infection. The promoter activities of the T3SS effector genes *exoT* and *exoS* were analyzed using a *lux*-based reporter. As shown in Figure 3E,F, in the presence of 5 mM sodium selenite, the expression of *exoS* and *exoT* was decreased significantly, indicating a decline of virulence. Taken together, these results suggested that 5 mM sodium selenite could inhibit virulence factor production of *P. aeruginosa* PAO1.

### 3.3. RNA-Seq Analysis Revealed the Global Response of P. aeruginosa in the Presence of 5 mM Sodium Selenite

**Figure 4:**
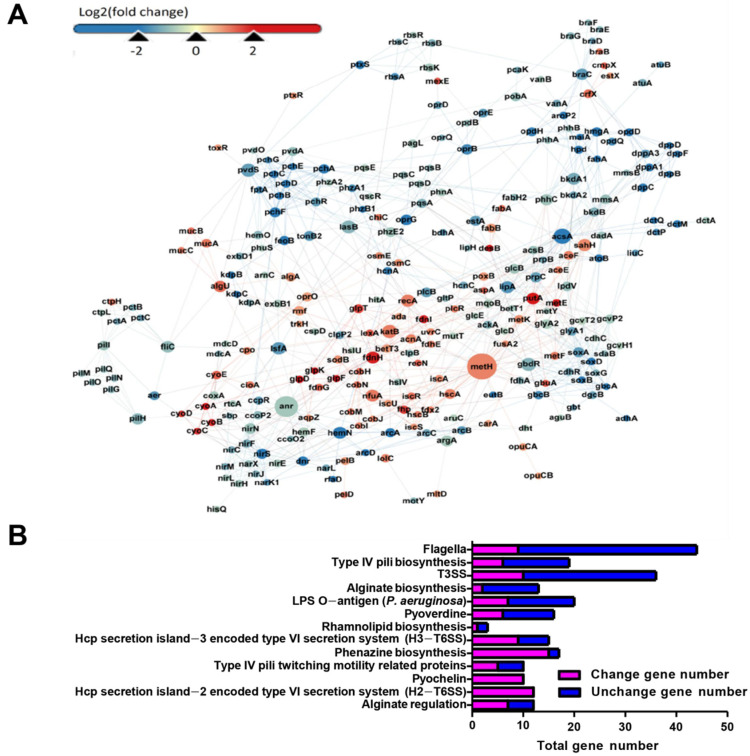
(**A**) Differential gene expression analysis diagram. Red represented high expression and blue represented low expression. The size of circles represented reliability, with bigger circles being higher reliability. (**B**) KEGG pathway analysis of DEGs. The top 13 classical pathways obtained through KEGG enrichment were shown.

To explore the global response of *P. aeruginosa* in the presence of 5 mM sodium selenite, RNA-seq analysis was performed. A total of 1023 DEGs were detected, with 368 up-regulated and 655 down-regulated (Figure 4A). Among them, the genes involved in the synthesis of pyocyanin, such as *phzA1*, *phzA2*, *phzB1*, and *phzE2* were down-regulated, and the expression levels of the genes involved in bacterial motility, such as *pilL*, *pilM, pilQ*, *pilO*, *pilG*, *pilH*, *and fliC* were significantly reduced. These data were consistent with the results of the phenotypes mentioned above (Figure 3). The down-regulated genes were closely related to the virulence of *P. aeruginosa*, indicating that sodium selenite might affect its pathogenicity.

Cluster analysis of COG was performed on all detected genes, and the results showed that the cluster of COG mainly was focused on amino acid transport and metabolism, energy production and conversion, inorganic ion transport and metabolism, lipid transport and metabolism, carbohydrates transport and metabolism, and signal transduction systems, and they accounted for 18.11%, 14.34%, 7.17%, 6.04%, 5.66%, and 5.28%, respectively (Figure S3). The results of cluster analysis of COG suggested that the presence of sodium selenite might inhibit the amino acid metabolism and carbohydrate metabolism, but improve the energy production of PAO1.

According to the results of the KEGG enrichment pathway, 13 significantly enriched pathways were selected. In each signal pathway, the total number of the genes and their expression changes were listed (Figure 4B). Among them, most DEGs were mainly enriched in the synthesis of pyocyanin and phenazines, type IV fimbria biosynthesis, flagella biosynthesis, alginate, and other extracellular polysaccharide synthesis pathways. In addition, a large number of DEGs were enriched in the type VI secretion system (T6SS) encoded by Hcp secretory Island-2 (H2-T6SS) and the Island-3 (H3-T6SS), two important virulence secretory systems of *P. aeruginosa*. In summary, the genes involved in the synthesis and secretion of various virulence factors were differentially expressed, indicating that sodium selenite had a significant effect on the virulence of *P. aeruginosa*.

### 3.4. Sodium Selenite Inhibited the Expression of Quorum Sensing Genes

**Figure 5:**
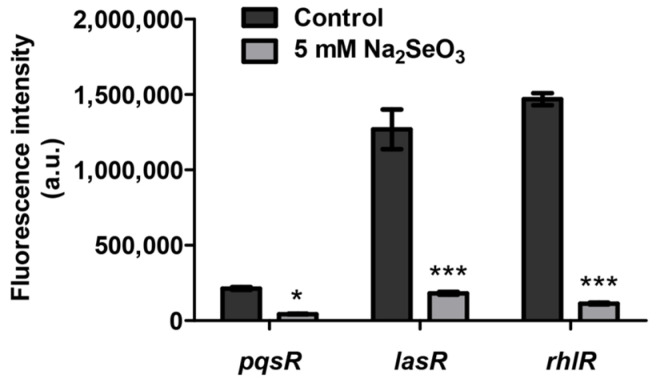
Sodium selenite inhibited the expression of quorum sensing genes *pqsR*, *lasR*, and *rhlR*. The expression levels of *pqsR*, *lasR,* and *rhlR* were detected by the GFP reporter vectors. Arbitrary fluorescence units (a.u.) were defined by the GFP intensity normalized with OD_600_. * *p* < 0.5, *** *p* < 0.001.

RNA-seq results showed that many genes related to quorum sensing were differentially expressed (Table 2). The *pqs* system genes such as *pqsABCDE* were significantly inhibited. The genes related to *rhl* system were also down-regulated. Previous studies have shown that *P. aeruginosa* activates the expression of many virulence genes by an intricate quorum sensing network. Therefore, in order to test whether sodium selenite inhibited virulence by affecting quorum sensing systems, the expression levels of *pqsR*, *lasR*, and *rhlR* were detected by the GFP reporter vectors. The result showed that the expression of these genes was decreased under the treatment of 5 mM sodium selenite, especially for the expression of *lasR* and *rhlR* (Figure 5). These results suggested that sodium selenite might reduce the virulence production of PAO1 by inhibiting its quorum sensing systems.

### 3.5. Sodium Selenite Increased the ROS Levels and the Expression of Oxidative Stress Related Genes

Among DEGs, numerous oxidative stress related genes were observed in the presence of 5 mM sodium selenite (Table 3), including *sodB*, *katB*, *katE*, *ahpC*, *ahpF*, and *ahpB*, which were regulated by two key regulation genes SoxR and OxyR. In addition, SoxR-regulated *zwf* (coding 6-phospho-glucose dehydrogenase for NADPH formation) and *acnA* (coding aconitase for NADPH formation) were also highly expressed. The genes involved in glutathione synthesis, such as *gshA* and *gshB*, were expressed at a low level. Taken together, the RNA-seq results confirmed that 5 mM sodium selenite might induce oxidative stress. The generated oxidative damage not only enhanced the bacterial susceptibility to antibiotics and inhibited the virulence, but also increased the expression of antioxidant genes in *P. aeruginosa* for self-protection.

**Figure 6:**
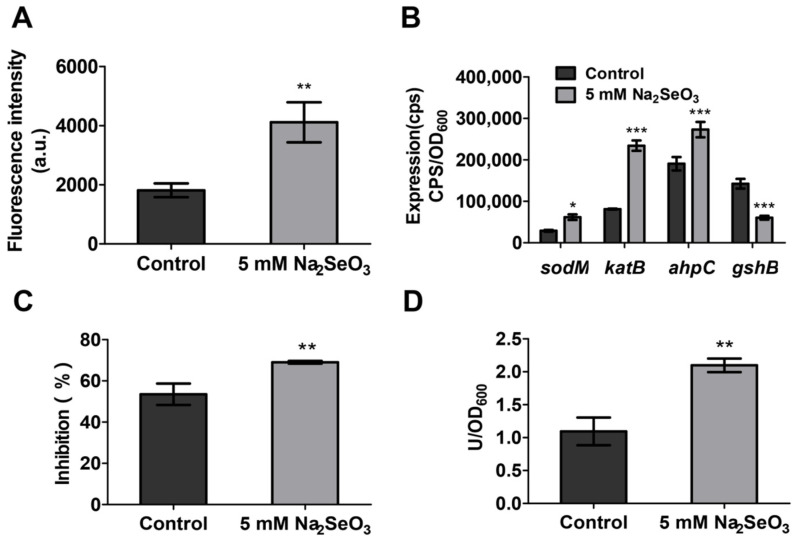
Sodium selenite induced oxidative stress. (**A**) Intracellular ROS levels were assessed by microplate reader using the probe Carboxy-H2DCFDA. (**B**) The expression levels of genes involved in oxidative stress were detected by using the reporter plasmid pMS402. (**C**) Measurement of the inhibition rate. It represented the hijacking of the O^2−^ and NBT reactions by SOD. (**D**) Measurement of total SOD activity. It was expressed as the enzyme activity unit U/OD_600_. All data were presented as mean ± SD of three separate experiments. * *p* < 0.5,** *p* < 0.01, *** *p* < 0.001.

It is reported that oxidative stress can affect the antibiotic susceptibility of pathogenic bacteria [32]. To investigate whether sodium selenite induced oxidative stress in *P. aeruginosa*, bacterial cells were labeled with a highly specific fluorescent probe, Carboxy-H2DCFDA, which is able to react with ROS and produce a fluorescent derivative 2′,7′-dichlorofluorescein (DCF) [33]. The result showed that the fluorescence intensity of cells treated with 5 mM sodium selenite was two-fold higher compared with the untreated cells (Figure 6A), indicating that sodium selenite increased the ROS level of *P. aeruginosa* cells.

In general, bacterial cells adopt defense mechanisms to scavenge ROS under the oxidative stress conditions. To evaluate the response of *P. aeruginosa*, the expressions of genes involved in the antioxidant system include superoxide dismutase (*SodM*)*,* catalase (*katB*), alkyl hydroperoxidase (*ahpC*), and glutathione synthetase (*gshB*) were examined. The results showed that the expression of most genes involved in oxidative stress was increased significantly in the presence of 5 mM sodium selenite except *gshB* (Figure 6B). Since the expression of *sodM* was induced by sodium selenite, the SOD activity was further tested. The inhibition of O^2−^ and the SOD activity were both increased in the sodium selenite group (Figure 6C,D). Overall, these results demonstrated that sodium selenite stimulated the production of ROS in *P. aeruginosa*, which induced the increase of antioxidant enzyme activity to eliminate the toxicity of ROS.

### 3.6. Sodium Selenite Reduced the Virulence of P. aeruginosa in Chinese Cabbage and D. melanogaster Infection Models

**Figure 7:**
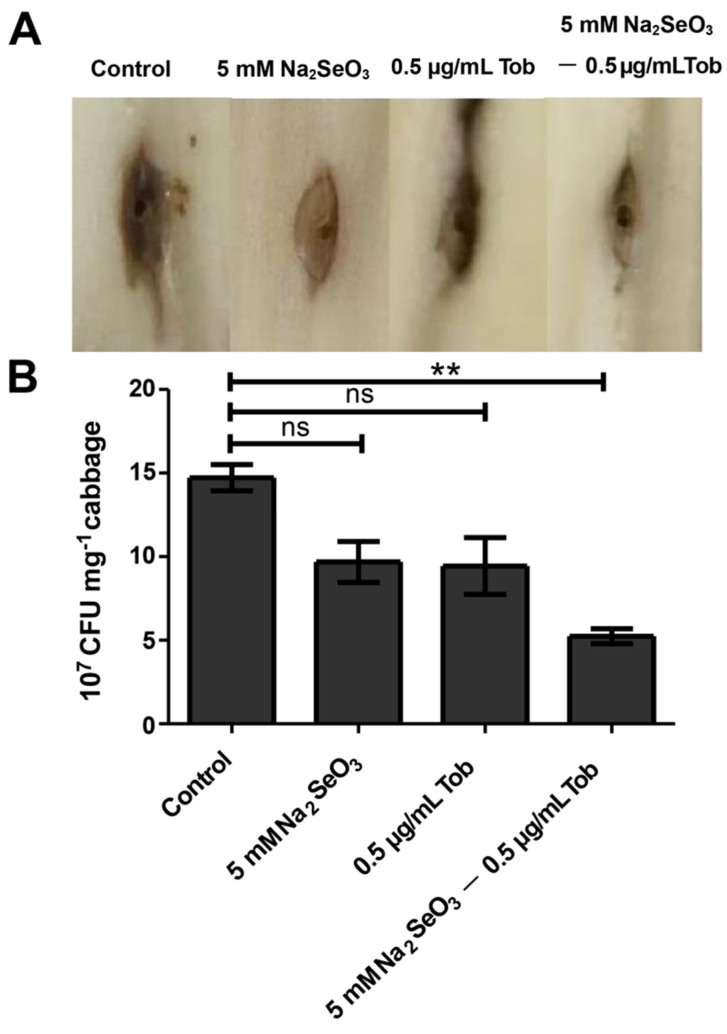
Sodium selenite in combination with tobramycin alleviated the virulence of PAO1. (**A**) The effects of 5 mM sodium selenite after the infection with PAO1 for 24 h, 36 h, and 48 h. (**B**) The numbers of bacterial cells (indicated as CFU) presented in 1 mg of cabbage midrib after injection for 2 days were shown. Error bars were calculated from three independent experiments, ** *p* < 0.01, compared with the untreated control group; ns, no significance.

The decrease of many virulence factors mentioned above indicated that the pathogenicity might be attenuated. Therefore, the damage of *P. aeruginosa* on Chinese cabbage leaves in the presence of sodium selenite was examined. The wild-type *P. aeruginosa* PAO1 strain inoculated into the middle rib of the Chinese cabbage was able to cause typical necrosis after inoculation for two days (Figure 7). When 5 mM sodium selenite or sub-MIC tobramycin was added, the necrosis of Chinese cabbage was weakened. However, when they were combined, the cabbage necrosis was significantly reduced (Figure 7A). In addition, the enumeration of *P. aeruginosa* cells proved the remarkable alleviation of infection (Figure 7B).

Considering that sodium selenite induced oxidative stress can reduce the pathogenicity of PAO1 in vitro, the effect of sodium selenite was further evaluated in vivo model. We used a *D. melanogaste*r infection model to detect the pathogenicity of PAO1. The results showed that the fruit-flies fed with *P. aeruginosa* PAO1 had a lower survival rate than those when 5 mM sodium selenite or sub-MIC tobramycin was added, although the combination did not significantly increase survival (Figure S4). However, this result also reflected that 5 mM sodium selenite showed no obvious toxicity to *D. melanogaster*.

## 4. Discussion

The emergence and spread of drug-resistant bacteria have become a major public health problem [34]. *P. aeruginosa* is a persistent pathogen, and it evolves multiple antibiotic resistances, such as inhibition of enzymatic action and decreased uptake of antibiotics via target molecular mutation and efflux pump alternation [35]. Sodium selenite, a basic nutrient element of organisms, is a good choice for developing new antibacterial agents [36]. Therefore, in this study the potential antibacterial mechanisms of sodium selenite against *P. aeruginosa* were investigated.

We examined the effect of different concentrations of sodium selenite on *P. aeruginosa* PAO1 growth. The results showed that sodium selenite had no significant impact on the growth of PAO1 when the concentration was lower than 5 mM (Figure S1). Therefore, sodium selenite of 5 mM was selected for antibacterial activity investigation, which did not affect the growth of PAO1 and avoided the generation of resistant strains.

The survival rate after the treatment of sodium selenite and the MIC value was obviously reduced compared to the antibiotic application group (Figure 1 and Figure 2). Further investigation demonstrated that the ROS level of the samples in the presence of sodium selenite was elevated and the bacterial anti-oxidant system was also induced (Figure 4), and therefore it was speculated that sodium selenite enhanced the antibiotics sensitivity of *P. aeruginosa* by inducing oxidative stress. Many antimicrobials exhibit bacteriological inactivation activity through ROS production [37], indicating that sodium selenite can be used as an adjunct drug of antibiotics to increase its killing effect and reduce the opportunity of drug-resistance bacterial occurrence.

Biofilm formation leads to strong resistance of bacteria to clinically common antibiotics and subsequently clinical refractory infection. Bacteria in the biofilm are reported to be 10 to 1000 times more resistant to antibiotics than the planktonic [38]. In this study, not only the biofilm formation was inhibited (Figure 2C,D), but also the eradication rate of the formed biofilm was increased when combined with antibiotics (Figure 2E,F). These results demonstrated that sodium selenite also increased the antibiotic susceptibility of bacteria in biofilm besides the planktonic, indicating a promising application potential of sodium selenite in biofilm inhibition and eradication.

The production of many virulence factors of *P. aeruginosa* PAO1 was inhibited by sodium selenite, including pyocyanin, rhamnolipid, and T3SS, and the motility of swarming, swimming, and twitching was all suppressed (Figure 3). These results might also lead to the decrease of bacterial antibiotics resistance in addition to pathogenicity. For example, pyocyanin, a virulence factor, is also involved in the biofilm formation and iron absorption of bacteria [39,40]. Rhamnolipid and motility are both related to bacterial biofilm formation [41,42].

Sodium selenite significantly inhibited the expression of quorum sensing related genes and significantly inhibited the virulence factors regulated by the quorum sensing. These results were validated by RNA-seq, indicating that sodium selenite might block quorum sensing system and reduce virulence factors production. These findings suggested that sodium selenite could be used as a quorum sensing inhibitor to reduce the virulence factor production and pathogenicity of *P. aeruginosa*. Quorum sensing inhibitors do not affect bacterial growth, but inhibit their virulence factors and reduce pathogenicity, while they do not cause bacterial drug resistance. Therefore, quorum sensing inhibitors are believed to generate weaker selection for antibiotic resistance than conventional antibiotics.

Sodium selenite promotes oxidative stress of *P. aeruginosa*. This bacterium has evolved various mechanisms to protect itself from oxidative stress and to survive in these adverse conditions. It is able to induce the antioxidant enzymes from the antioxidant system to balance ROS, such as SOD, catalase (CAT), and alkyl hydroperoxidase (AHPC), which catalyze the decomposition of O^2−^, H_2_O_2,_ and OH^−^ in bacterial cells, respectively, to resist the toxicity of ROS. In addition, this bacterium also produces a large number of antioxidant molecules, such as GSH. GSH, the first antioxidant in tissue defense, protects cells and tissues from oxidative damage by eliminating or blocking the production of ROS [43]. GSH is oxidized under oxidative stress, and the decrease of glutathione level is one of the general characteristics of oxidative stress [44]. Our data showed that the expression levels of these antioxidant enzyme genes *katB*, *sodB*, and *ahpC* were increased while the expression level of *gshB* was decreased (Figure 6B). In addition, the activity of total SOD in PAO1 was increased under the treatment of sodium selenite (Figure 6C,D). Relevant studies have confirmed that when *P. aeruginosa* PAO1 was exposed to exogenous oxidative stress, the expression of antioxidant enzyme genes *katB* and *ahpC* increased [45]. It is reported that after induction of *P. aeruginosa* by phytol isolated from aster yomena, ROS content in bacterial cells was increased, and the imbalance of the antioxidant defense system resulted in a decrease of glutathione GSH [46]. GSH also plays an important role in the oxidative stress, bacterial toxicity, and biofilm formation of *P. aeruginosa* [47]. It was confirmed that after *gshB* gene deletion mutation, the production of pyocyanin and pyoverdine decreased, and the cell swimming and twitching mobility decreased. These phenomena were consistent with our results. Taken together, these findings help to explain the antibacterial mechanism of sodium selenite.

The effect of sodium selenite on the pathogenicity of *P. aeruginosa* was tested by using a Chinese cabbage infection model (Figure 7). It is found that sodium selenite had a certain antibacterial effect, and alleviated the pathogenicity of *P. aeruginosa*. In addition, for the plant itself, sodium selenite may be a nutritional enhancer. Many studies have shown that foliar spray of sodium selenite can promote plant growth and improve plant nutrient [48]. Considering that sodium selenite reduced the toxicity of *P. aeruginosa* in Chinese cabbage infection, we hypothesized that this antibacterial activity of sodium selenite could also provide an advantage for animal immune defense. The results of the *D. melanogaster* infection model confirmed that sodium selenite had a certain inhibitory effect on the pathogenicity of PAO1, although the combination of antibiotics did not achieve a good antibacterial effect (Figure S4). This may be due to the fact that *D. melanogaster* itself metabolizes sodium selenite, and it is worthy to be explored in future work.

Currently, a new antimicrobial strategy is to target the key toxicity of pathogens, aiming to reduce the pathogenicity rather than the growth of microorganisms. The molecules that target virulence factors of pathogens are neither bacteriostatic nor bactericidal, and the probability of bacterial resistance development against these molecules is minimal due to less selection pressure [49]. Therefore, sodium selenite can be used as a potential alternative therapeutic agent or an adjunct therapy in combination with currently practiced antibiotic regimens to reduce disease progression and antibiotic use. However, it needs to be paid attention that although selenium is an essential nutrient element for the human body, inorganic sodium selenite has high toxicity [50], and its application in human antibacterial therapy still needs further exploration. Selenium nanoparticles (SeNPs) and organic selenium, such as selenium methionine, may be used for antibacterial treatment. SeNPs has the ability to destroy microbial biofilm formation [51,52] and has been used as an antibacterial agent, antioxidant and anticancer agent in medicine due to its good antibacterial and antioxidant properties, and has the potential to become a new therapeutic drug and selenium nutritional supplement [53,54].

## 5. Conclusions

In this work we investigated the effects of sodium selenite on the antibiotic susceptibility and virulence related phenotype of *P. aeruginosa* PAO1. It was found that less than 5 mM sodium selenite did not obviously affect the bacterial growth, but decreased the MIC, MBIC, and MBEC values of the antibiotics tested. Further investigation found that sodium selenite induced oxidative stress and increased ROS accumulation and antioxidant enzyme activity, which might be the reasons for antibiotic susceptibility changes. In addition, sodium selenite inhibited bacterial virulence and reduced pathogenicity by disturbing the expression of quorum sensing related genes. Taken together, the results demonstrated the influence of sodium selenite on the physiology of *P. aeruginosa*, and indicated a promising application potential of sodium selenite for *P. aeruginosa* infection, especially for the bacteria within biofilms.

## Figures and Tables

**Table 1:** Bacterial strains, plasmids, and primers used in this study.

Names ^1^	Relevant Characteristics	Accession Number of the Involved Genes ^2^	Source
Strains			
*E. coli* DH5α	*F^–^ φ80lacZ* Δ*M15* Δ*(lacZYA-argF)U169 recA1 endA1 hsdR17 (rk, mk^+^) phoA supE44 thi-1 gyrA96 relA1 tonA*	-	Tiangen
*P. aeruginosa* PAO1	*P. aeruginosa* wild type	-	this lab
PAO1 (pHERD20T- *pqsR*-gfp)	PAO1 containing *pqsR*-gfp reporter plasmid pHERD20T (Cb^r^)	879994	this lab
PAO1 (pHERD20T- *lasR*-gfp)	PAO1 containing *lasR*-gfp reporter plasmid pHERD20T (Cb^r^)	881789	this lab
PAO1 (pHERD20T- *rhlR*-gfp)	PAO1 containing *rhlR*-gfp reporter plasmid pHERD20T (Cb^r^)	878968	this lab
Plasmids			
CTX-*exoS*	Integration plasmid, CTX6.1 with a fragment of pKD-*exoS* containing *exoS* promoter region and *luxCDABE* gene; Kn^r^, Tmp^r^, Tc^r^	879837	this lab
CTX-*exoT*	Integration plasmid, CTX6.1 with a fragment of pKD-*exoT* containing *exoT* promoter region and *luxCDABE* gene; Kn^r^, Tmp^r^, Tc^r^	878350	this lab
pMS402	Expression reporter plasmid carrying the promoterless *luxCDABE* gene; Kn^r^, Tmp^r^	-	[21]
pKD-*sodM*	pMS402 containing *sodM* promoter region; Kn^r^, Tmp^r^	881040	this study
pKD-*katB*	pMS402 containing *katB* promoter region; Kn^r^, Tmp^r^	881120	this study
pKD-*ahpC*	pMS402 containing *ahpC* promoter region; Kn^r^, Tmp^r^	879431	this study
pKD-*gshB*	pMS402 containing *gshB* promoter region; Kn^r^, Tmp^r^	878223	this study
Primers	Sequence (5′→3′)		Restriction sites
*sodM*-S	TATCTCGAGTGGTCGAGTCGATGATGG	881040	*Xho* I
*sodM*-A	ATAGGATCCGACATGGCAACCTCACCA	881040	*Bam* HI
*katB*-S	GATCTCGAGTCACTCCCTGTATTTCGC	881120	*Xho* I
*katB*-A	GTAGGATCCAGGGTTCATGGAAGAGCT	881120	*Bam* HI
*ahpC*-S	GATCTCGAGGGCAGGTTCTTCGATTAG	879431	*Xho* I
*ahpC*-A	GTTGGATCCTCAGGGACATCAGTCGTT	879431	*Bam* HI
*gshB*-S	GATCTCGAGCACTTTCAAACCGTCGGA	878223	*Xho* I
*gshB*-A	GAAGGATCCCGTACGCTCATGGGAATT	878223	*Bam* HI

^1^*E. coli* DH5α was purchased from Beijing Tiangen Biochemical Technology Co. Ltd., Beijing, China. *P. aeruginosa* wild-type strain PAO1, the wild-derived strains, pKD-*sodM*, pKD-*katB*, pKD-*ahpC* and pKD-*gshB* were stored in this laboratory (Key Laboratory of Resources Biology and Biotechnology in Western China, Ministry of Education, College of Life Sciences, Northwest University, Xi’an, China). ^2^ ID of the involved genes was displayed; -, not applicable. The underlines indicated restriction enzyme sites.

**Table 2:** Differential expression genes involved in quorum sensing system.

Locus Tag	Gene Name	Fold Change	*P*_adj_	Product
PA0996	*pqsA*	0.3252	1.01 × 10^−47^	PqsA
PA0997	*pqsB*	0.3648	3.40 × 10^−38^	PqsB
PA0998	*pqsC*	0.3866	6.23 × 10^−32^	PqsC
PA0999	*pqsD*	0.3954	1.53 × 10^−33^	3-oxoacyl-[acyl-carrier-protein] synthase III
PA1000	*pqsE*	0.4951	3.45 × 10^−18^	quinolone signal response protein
PA1432	*lasI*	0.8302	9.27 × 10^−2^	autoinducer synthesis protein LasI
PA1430	*lasR*	0.9847	8.73 × 10^−3^	transcriptional regulator LasR
PA3476	*rhlI*	0.6924	2.36 × 10^−5^	autoinducer synthesis protein RhlI
PA3477	*rhlR*	0.7597	3.37 × 10^−4^	transcriptional regulator RhlR

**Table 3:** Differential expression genes involved in oxidative stress.

Locus Tag	Gene Name	Fold Change	*P*_adj_	Product
PA2273	*soxR*	1.0377	7.98 × 10^−1^	SoxR
PA3183	*zwf*	1.1942	1.68 × 10^−2^	Glucose-6-phosphate 1-dehydrogenase
PA4366	*sodB*	2.2508	7.69 × 10^−23^	superoxide dismutase
PA1562	*acnA*	2.5174	1.32 × 10^−40^	aconitate hydratase 1
PA5344	*oxyR*	0.9082	2.24 × 10^−1^	OxyR
PA4613	*katB*	2.6860	2.03 × 10^−39^	catalase
PA2147	*katE*	1.9478	2.38 × 10^−4^	catalase HPII
PA2185	*katN*	1.8293	3.23 × 10^−3^	non-heme catalase KatN
PA0139	*ahpC*	1.1545	4.69 × 10^−2^	alkyl hydroperoxide reductase subunit C
PA0140	*ahpF*	1.6734	3.49 × 10^−13^	alkyl hydroperoxide reductase subunit F
PA0848	*ahpB*	2.6370	2.96 × 10^−28^	alkyl hydroperoxide reductase
PA2025	*gor*	1.3427	7.19 × 10^−5^	glutathione reductase
PA5203	*gshA*	0.8096	4.88 × 10^−3^	glutamate--cysteine ligase
PA0407	*gshB*	0.7360	6.81 × 10^−5^	glutathione synthetase
PA4210	*phzA1*	0.1995	1.20 × 10^−9^	probable phenazine biosynthesis protein
PA1899	*phzA2*	0.4405	1.28 × 10^−2^	probable phenazine biosynthesis protein
PA4214	*phzE1*	0.4272	1.17 × 10^−12^	phenazine biosynthesis protein PhzE
PA1903	*phzE2*	0.4272	1.17 × 10^−12^	phenazine biosynthesis protein PhzE
PA3812	*iscA*	2.2484	1.52 × 10^−28^	probable iron-binding protein IscA
PA3813	*iscU*	2.1258	4.92 × 10^−28^	probable iron-binding protein IscU
PA4615	*fprB*	2.8740	1.41 × 10^−46^	FprB
PA2356	*msuD*	1.9334	2.24 × 10^−1^	methanesulfonate sulfonatase MsuD

## Data Availability

All the data are available within the article or Supplementary Materials.

## Supplementary Materials

The following are available online at https://www.mdpi.com/article/10.3390/antiox10121873/s1, Figure S1: Sodium selenite with the concentrations between 0.5–5 mM did not obviously affect the growth of P. aeruginosa PAO1, Figure S2: Sodium selenite inhibited the bacterial motilities, Figure S3: The COG enrichment analysis of the differentially expressed genes, Figure S4. Sodium selenite enhances the survival rate of Drosophila melanogaster, Table S1: The values of MIC, MBIC, and MBEC in the different combinations of antibiotics and sodium selenite
