# Supplementary material for: Sodium Selenite Enhances Antibiotics Sensitivity of Pseudomonas aeruginosa and Deceases Its Pathogenicity by Inducing Oxidative Stress and Inhibiting Quorum Sensing System
Source: Antioxidants (Basel). 2021 Nov 24;10(12):1873. doi: 10.3390/antiox10121873 (PMC8698442; doi:10.3390/antiox10121873)
Supplement: Supplementary file 1 [file antioxidants-10-01873-s001.zip › antioxidants-1455661-supplementary.pdf]

Supporting information

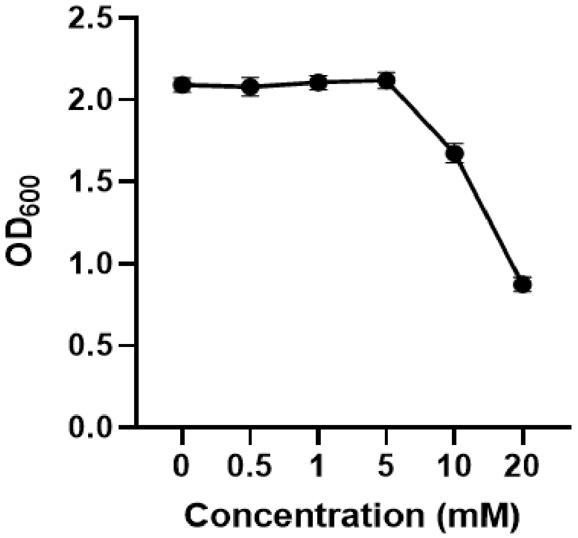

Figure S1. Sodium selenite with the concentrations between 0.5-5 mM did not obviously affect the growth of *P. aeruginosa* PAO1. PAO1 was cultured in the LBNS broth with different concentrations of sodium selenite for 12 h.

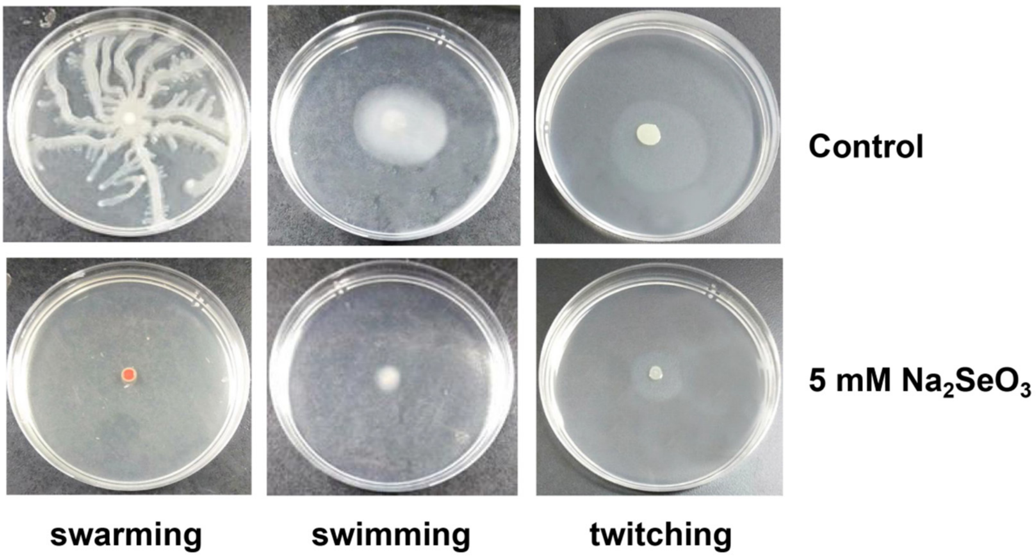

Figure S2. Sodium selenite inhibited the bacterial motilities. Swarming motility, 2 μL of overnight cultures were spotted onto swarming plates. After the plates were incubated at 37 °C for 12 h, the images were captured. Swimming motility, 2 μL of overnight cultures were spotted onto swimming plates. After the plates were incubated at 30 °C for 12 h, the images were captured. Twitching motility was performed by stab-inoculating bacteria through the thin LBNS agar plates at 30 °C for 24 h.

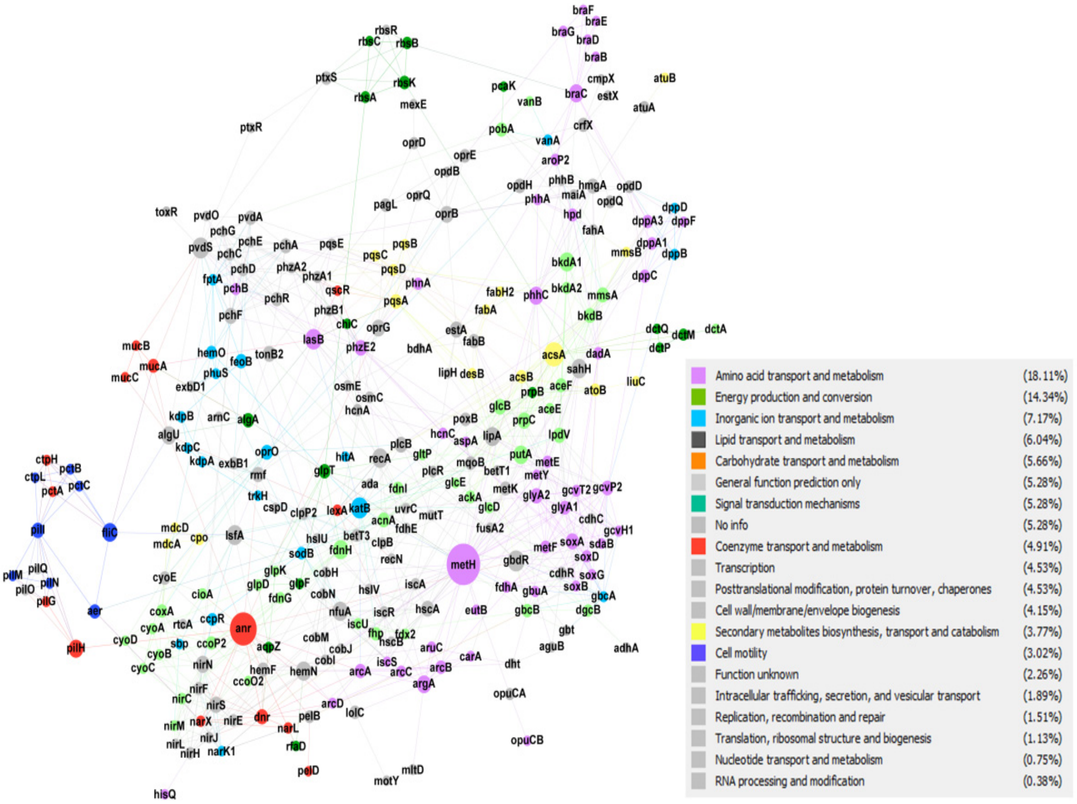

Figure S3. The COG enrichment analysis of the differentially expressed genes. The DEGs were classified into 20 primary COG categories and labeled the proportions for each category.

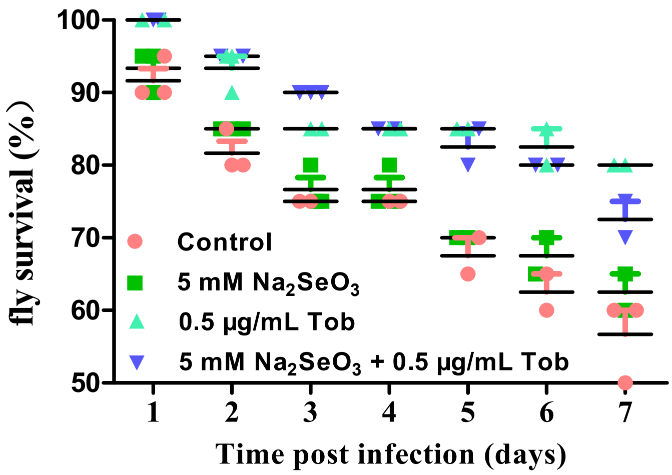

Figure S4. Sodium selenite enhances the survival rate of *Drosophila melanogaster*. The percentages of *D. melanogaster* survived at each time point were recorded after being fed with the *P. aeruginosa* PAO1. Each point of the survival curves is the average of triplicate experiments expressed as means + SD.

Table S1. The values of MIC, MBIC, and MBEC in the different combinations of antibiotics and sodium selenite

Treatments	Antibiotics[1]	Sodium selenite (0 mM)	Sodium selenite (1 mM)	Sodium selenite (5 mM)
MIC	gentamicin	4	4	2
	tobramycin	2	1	0.5
MBIC	gentamicin	4	2	1
	tobramycin	4	2	0.5
MBEC	gentamicin	2	2	1
	tobramycin	0.5	0.5	0.25

[1] The unit of antibiotic concentration was μg/mL.
